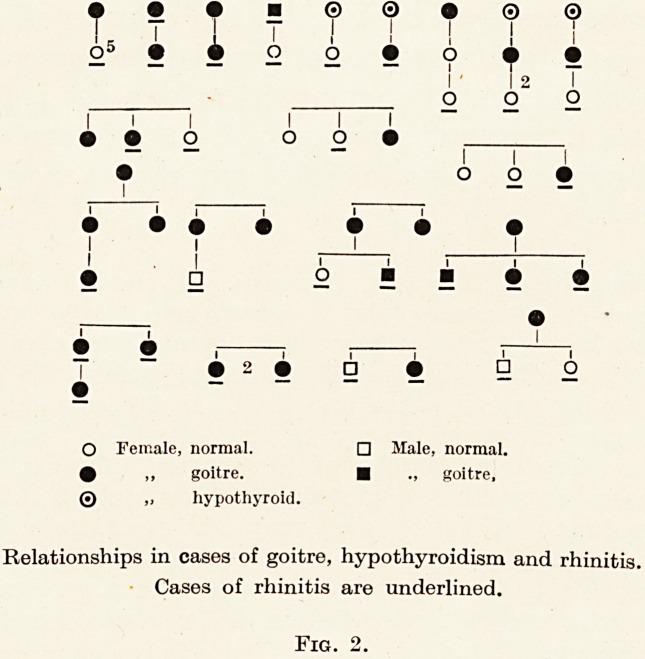# The Association between Goitre and Atrophic Rhinitis
*A paper read before the Bristol Medico-Chirurgical Society on Wednesday, 10th January, 1934, when fifteen patients were shown to demonstrate the clinical condition described.


**Published:** 1934

**Authors:** E. Watson-Williams

**Affiliations:** Surgeon in Charge, Ear, Nose and Throat Department, Bristol Royal Infirmary


### The Bristol Medico-Chirurgical Journal

*" Scire est nescire, nisi id me
Scire alius sciret."*

SUMMER, 1934.

### THE ASSOCIATION BETWEEN GOITRE AND ATROPHIC RHINITIS.*

BY

E. Watson-Williams, M.C., Ch.M., F.R.C.S.,
*Surgeon in Charge, Ear, Nose and Throat Department,
Bristol Royal Infirmary.*

The association between the endocrine glands and the nose has long been recognized. One may instance the turbinal engorgement and increased secretion following sexual excitement, the so-called " honeymoon catarrh." Again, many observers, especially on the Continent, have believed that there is a direct connection between goitre and atrophic rhinitis. It was this, in particular, that it was resolved to examine when this investigation was begun exactly seven years ago, in February, 1927, both because the thyroid is the only endocrine gland that lends itself readily to clinical examination, and also because this is the only gland-substance that can be easily administered by mouth with a reasonable certainty of foreknown results.

* A paper read before the Bristol Medico-Chirurgical Society on Wednesday, 10th January, 1934, when fifteen patients were shown to demonstrate the clinical condition described.

The classical picture of atrophic rhinitis is that of an adult, more often than not female, surrounded by a characteristic sour fetor which gives the condition its alternative name of "ozœna," but having herself lost all sense of smell; whose nasal chambers are much wider than normal, without turbinal bodies, and occupied by masses of sour, green, dry secretion. And it is said that the "disease" is becoming rare. No doubt the recognition of sinus infection and the better treatment of syphilis (the stink from a necrotic vomer must be experienced to be believed) have eliminated many cases that would formerly have filled this category. But if by atrophic rhinitis we mean only a condition of abnormal patency of the nose due to atrophy of the turbinal bodies and bones, there are still to be found many cases, although perhaps relatively few of them present the extreme degree of atrophy that was once considered necessary for inclusion under this head.

In the normal nose, when we examine the interior by means of a forehead mirror or light, we see that the turbinal bodies, and especially the inferior, do not quite touch the septum, but the narrow space between fades into the obscurity at the back of the nose where details cannot be made out. (Fig. 1.) Abnormally, however, it may be possible to see past the turbinals quite back into the naso-pharynx, and to discern the red colour of the back wall, and the movements

Normal.

A1.

A2.

A3.

A4.

A5.

FIG. 1.

there which accompany deglutition. This may be taken as the least evidence of definite atrophy, and we have called this A2. A1 is reserved for conditions where the turbinal bodies are obviously smaller than normal, but the test just described does not apply. A3 means definite, unequivocal atrophy; the inferior turbinals are very much smaller than normal, so that the middle turbinals (which are generally less affected in these cases) stand out prominently in the upper part of the nose near the septum. A4 means that the inferior turbinals are practically absent, and the outward curve of the vomer at the back of the nose is well seen; the middle turbinals, also, are clearly smaller than usual, and one can see the front of the sphenoid. A5 is the most extreme degree, in which hardly any vestiges of turbinals can be seen, and generally the sphenoidal ostium is visible from the nostril.

Observing a large series of these cases, and watching the progress in some over several years, one conceives the atrophic process as affecting primarily the soft parts, especially the erectile tissue (thus explaining why the inferior turbinals are most manifestly affected in the earlier stages). But later on there is a gradual absorption of the actual turbinal bones, so that the picture of more advanced atrophy is seen; it seems impossible to draw a hard-and-fast line between one stage and another. Of course, when suppurative rhinitis or syphilitic disease attacks the turbinal bones, a rapid and severe destruction is produced, and possibly then a different pathology accounts for the atrophy of the soft parts.

The mucous membrane in the early stages is pale, thin and "poor-looking." It appears as if ciliary activity was lost. Later it becomes dry, and in about half of these cases (in all groups) small scabs of drying secretion lie on the surface. In another type there is a very remarkable excess of glairy, "milky-watery" mucoid secretion, and this, as is shown in the table, is not confined to those cases where the atrophy is least, but may occur in even the most extreme atrophy. Purulent secretion is not very common, apart from sinus disease, or in association with crusting. Actual gross crusts are naturally most often seen in the cases with very wide nasal chambers; drying, rather than any specific infective or other cause, seems to determine their presence. Where the nasal passage is very wide the covering is a squamous epithelium, with very little mucous secretion.

In addition to the abnormal patency and nasal secretions, other features to which attention was directed in the cases to be described below were (1) anosmia, loss or diminution of sense of smell; (2) fetor, an unpleasant odour perceived by the observer, generally the typical sour smell of mucus, but not always associated with crusts or excess of mucoid secretion; (3) cacosmia, an unpleasant smell observed by the patient; (4) perforation of the septum; (5) secondary drying or other effects in the pharynx or larynx; (6) the Wassermann reaction, and other evidence of syphilis; (7) the condition of the accessory sinuses. Some of the more important of these are indicated in the table (see pages 94 and 95).

The clinical material consists of 473 cases: (*a*) all cases on my index as "syphilitic rhinitis," (*b*) all cases of atrophic rhinitis, and (*c*) all cases of goitre seen during the seven years. All cases of traumatic origin, whether accidental or surgical, are excluded. In the table these have been divided into various groups according to the probable causative factor, and the age and sex distribution, degree of atrophy, etc., are indicated for each group (bold figures). Neglecting for the moment 31 cases in which the atrophy was slight, there are 334 cases of atrophic rhinitis, 188 "definite," 109 severe, 37 either definite or severe. When we endeavour to assign cases to their groups some difficulty arises; for example, among the "goitre" group are 5 cases in which symptoms were ascribed to an acute specific fever, and 3 cases of severe anæmia. Any patient who definitely or probably had had syphilis is included in that group, although there were several with goitre. We will leave the "goitre" group for discussion later, and ignore the small groups associated with anæmia (which, as a group, closely resembles the exanthem group), lupus, and the "miscellaneous" that have not been easily classified.

Syphilis: 125 patients, 80 males. Four-fifths of all these patients were above the age of 30, and in each age-group males are nearly twice as frequent as females, probably from unequal incidence of the original disease. It must be remembered that syphilitic patients with normal noses or those indexed under "polypi," "sinus disease" and the like are not included, owing to the labour involved in searching for them; also, many patients with minor degrees of atrophy may have been overlooked. But, even allowing for this, a remarkable feature of this group is the large proportion of patients with *severe* atrophy, 53 being graded A4, 27 A5. 83 patients had fetor, among whom 63 showed extreme atrophy, 37 perforation of the septum, 54 had crusts in the nose, 47 cacosmia, 67 anosmia. Among the 42 patients without fetor only 17 showed extreme atrophy, 5 perforation of the septum, only 5 had nasal crusting, 11 cacosmia, 23 anosmia (see p. 101). Most of the patients with purulent discharge had cacosmia. But even among those with extreme atrophy

On the left is shown the suggested ætiology with age and sex distribution: on the rig
The centre portion is

| Ætiological Group. | Sex. | Age Group. 0—14 | 15—29 | 30—44 | 45— | Complaint. Goitre | Catarrh. | Fetor. | Dry Throat. | Deafness. | Pain. | | | Olfaction dim. | Al. N |
|---|---|---|---|---|---|---|---|---|---|---|---|---|---|---|---|
| Syphilis .. 125[a] | M | 10 | 7 | 42 | 21 | | | | | | | mucus | 28[a] | 14 | |
| | | | | | | | | | | | | dry | 16 | 11 | |
| | F | 6 | 4 | 20 | 15 | | | | | | | pus | 22 | 17 | |
| | | | | | | | | | | | | crusts | 59 | 48 | |
| Exanthemata 24[e] | M | 7 | 4 | | | | 13 | 2 | | 2 | | mucus | 5 | 0 | |
| | F | 8 | 5 | | | | | | | | | dry | 6 | 0 | |
| | | | | | | | | | | | | crusts | 13 | 0 | |
| Simple Goitre 73[d] | M | 2 | 4 | 2 | | 8 | 23 | 9 | 19 | 4 | 4 | mucus[b] | 29 | 3 | 3 |
| | F | 20 | 26 | 15 | 4 | | | | | | | dry | 44 | 8 | 8 |
| Hypothyroidism 42 | M | 1 | | 1 | | | 10 | | 18 | 8 | 3 | mucus | 11 | 2 | 1• |
| | F | 2 | 7 | 2 | 29 | | | | | | | dry | 31 | 18 | 3 |
| Relatives have Goitre .. 18 | M | 3 | | | | | 11 | 1 | | 1 | 1 | mucus | 10 | 2 | 2 |
| | F | 10 | 3 | 1 | 1 | | | | | | | dry | 8 | 4 | |
| Composite "Goitre" Group 133[b] | M | 6 | 4 | 3 | | 8 | 44 | 10 | 37 | 13 | 8 | mucus | 49 | 7 | 6• |
| | F | 32 | 36 | 18 | 34 | | | | | | | dry[b] | 74 | 23 | 11 |
| | | | | | | | | | | | | crusts | 8 | 7 | |
| Clinically Similar 55 | M | 15 | 2 | 2 | | | 27 | 5 | 6 | 3 | 4 | mucus | 24 | 3 | 3 |
| | F | 15 | 16 | 5 | | | | | | | | dry | 19 | 2 | 2• |
| | | | | | | | | | | | | crusts | 12 | 7 | |
| Toxic Goitre[k] 26 | M | 0 | 2 | 3 | | | | | | | | mucus | 5 | 0 | |
| | F | 1 | 8 | 8 | 4 | | | | | | | dry | 6 | 0 | 2 |
| Lupus .. .. 15 | | | | | | | | | | | | | | | |
| Anæmia[h] .. 11 | | | | | | | | | | | | | | | |
| Miscellaneous 22 | | | | | | | | | | | | | | | |
| Total[d] 435 | | | | | | | | | | | | | | | |

*a* Including 12 cases of hypertrophy. *b* Including 3 cases of hypertrophy.
*e* Five of these cases have been counted also in the "goitre" groups.
*p* One case with pus. • One case with cacosmia. N No fetor. F Fetor.

…r each degree of atrophy, the number of cases (in heavy type) and of cures (in italics).
…alysis of symptoms and signs.

| …grees of Atrophy. | | | | | | | Treatment. | | | | | | | | | | | | |
|---|---|---|---|---|---|---|---|---|---|---|---|---|---|---|---|---|---|---|---|
| A2. | A3. | | A4, 5. | | Total fetor. | Perforation septum. | A1. | | A2. | | A3. | | A4, 5. | | Totals. | | | Given. | |
| F | N | F | N | F | | | Total Cases. | Cured. | Total Cases. | Cured. | Total Cases. | Cured. | Total Cases. | Cured. | Cases | Cured. | Treated | Iodine. | Thyroid |
| | 3 | 5 | | 7 | 12 | | | | 3 | | 30 | | 80 | | | | | | |
| | 4 | 2 | 4 | 6 | 8 | | | | | | | | | | | | | | |
| | 3 | 2 | 8 | 9 | 11 | 8 | | | | | | | | | | | | | |
| 2 | | 11 | 5 | 41 | 54 | 34 | | | | | | | | | | | | | |
| 2 | 2 | 1· | | | 3 | | | | 4 | | 9 | | 11 | | | | | | |
| | 1 | 2 | | 2·· | 4 | | | | | | | | | | | | | | |
| 1 | 1· | 2·· | | 9··· | 12 | 2 | | | | | | | | | | | | | |
| 3·· | 8 | 2·· | 1 | | 6 | | 13 | *5* | 25 | *7* | 29 | *16* | 3 | *0* | 70 | *28* | 55 | 27 | 28 |
| 3·· | 12···$_{p}$ | 7···$_{pcc}$ | 2 | | 11 | 2 | | | | | | | | | | | | | |
| | 4 | 4·· | | | 4 | | 8 | *6* | 7 | *3* | 23 | *15* | 4 | *1* | 42 | *25* | 36 | 3 | 33 |
| 1[c] | 12[c] | 3 | 4··[c] | | 8 | | | | | | | | | | | | | | |
| | 7 | | | | 0 | | 2 | *1* | 3 | *2* | 12 | *6* | 1 | *0* | 18 | *9* | 14 | 3 | 11 |
| 1[c] | 1 | 4[cc]$_{p}$ | | 1·$_{p}$ | 6 | | | | | | | | | | | | | | |
| 3·· | 18 | 6···· | 1 | | 10 | | 23 | *12* | 35 | *12* | 64 | *37* | 8 | *1* | 130 | *62* | 105 | 33 | 72 |
| 3· | 25···$_{p}$ | 9··$_{pp}$ | 5·· | 1[p] | 16 | | | | | | | | | | | | | | |
| 2· | 1 | 4· | 1 | | 8 | 2 | | | | | | | | | | | | | |
| 1 | 13 | 2 | 2 | | 3 | | 6 | *5* | 7 | *3* | 32 | *18* | 10 | *5* | 55 | *31* | 48 | 10 | 38 |
| 1 | 7· | 3··$_{pp}$ | 2 | 2· | 6 | | | | | | | | | | | | | | |
| | 2· | 5· | | 4 | 10 | | | | | | | | | | | | | | |
| | 2 | | 1 | | 0 | TOTAL | 29 | *17* | 42 | *15* | 96 | *55* | 18 | *6* | 185 | *93* | 153 | 43 | 110 |
| | 2 | | | | 0 | | | | | | | | | | | | | | |

c One case with crusts.
d In addition, there were 38 patients with no nasal abnormality.
h Including 4 cases counted in goitre and similar groups.
k Including 9 cases of hypertrophy and 6 normal.

and fetor there were 7 patients with excess of mucoid secretion but no crusts or pus. The Wassermann reaction was positive in 64 cases, negative in 25.

Exanthemata: 24 cases, 11 males. 5 cases are included which are also included among the "goitre" and similar groups. The sexes are equal, and all the patients belong to the younger age-groups. 11 cases showed severe atrophy, 2 perforation of the septum (the greatest care was taken to be sure that these were not syphilitic), and 13 crusting. Fetor was present in all the cases with severe atrophy, and in all but 5 of the remainder; cacosmia occurred in half the cases in each degree of atrophy, except A2, where there was none. No patient had anosmia. 10 patients had a negative Wassermann reaction. The sinuses were examined in 7, and found infected in 1 only (A2, fetor, mucoid secretion, no cacosmia). Measles was blamed in 8 cases, scarlet fever in 12, diphtheria in 4. (I was not aware until beginning this analysis how important were the exanthemata in this connection. There is no doubt that the group ought to be larger; for example, some of those classed as "clinically similar to goitre" should really appear here, but only those cases are included in which the *patient volunteered* that symptoms dated from such a disease.)

These two groups may be regarded as showing the result of severe destructive lesions on the nose. The age incidences are what one would expect; in the exanthem group the sexes are equal, in syphilis males predominate. In both groups a large proportion of the cases shows severe atrophy, about one-quarter have excess of mucoid secretion, and one-half suffer from crusting in the nose. Anosmia is not seen in the exanthem group, but affects two-thirds of the syphilis group. Cacosmia and fetor are far more frequent in the cases with great atrophy and especially in those with crusts.

If there is a real association between atrophy and goitre, one would expect patients with goitre or similar evidence of thyroid mal-function to show a like definite symptomatology, age and sex incidence, degree of atrophy, frequency of crusting, fetor, cacosmia, etc. The presence of a simple goitre (with which is included simple thyroid adenoma) is taken as evidence of deficiency in thyroid activity or of the quality of the secretion. But the exact correlation of goitre with atrophy is not clear; a large or old-standing goitre does not imply severe atrophy, nor *vice versa*. In the same way, it is sometimes difficult to assign a given case to "simple" or to "toxic goitre"—the features are intermingled. All that can be claimed is that the presence of a goitre is evidence of some disturbance of thyroid function, and is often associated with nasal disease. The symptomatology, age and sex incidence, degrees of atrophy, and other features of the sub-groups composing this group are shown in the table.

Simple goitre: 111 cases, 12 males. Of these 38 had no nasal signs or symptoms, and 8 complained only of goitre, although the nose was abnormal. 1 patient was gravely anæmic, 2 ascribed the trouble to exanthemata. 5 were already taking thyroid when first seen. In 70 cases showing some degree of atrophy only 3 were classified "severe." 2 patients showed perforation of the septum. Over a third of the cases had excess of mucoid secretion, only 2 suffered from crusting. 17 had fetor, evenly divided between the moist and dry. Cacosmia was more frequent among the dry cases, but neither fetor nor cacosmia was much more common in the more severely atrophic than in the less. 6 had negative Wassermann reaction.

Hypothyroidism (including 5 cases of classical myxœdema): 22 patients, 2 males. 7 of these were thus classified on account of amenorrhœa, etc. In 2 cases the complaint followed an operation for simple goitre. Only 4 showed severe atrophy; none had crusting or cacosmia, but 6 had fetor. Wassermann reaction negative in 8.

Menopause rhinitis: 20 females. The distinction between these cases and the last was in many cases very doubtful, and the two are shown as one sub-group. One was gravely anæmic, another blamed pneumonia as the origin of nasal disease. In 3 patients the menopause was due to surgical measures. 3 patients showed severe atrophy, none crusting, 6 fetor. Wassermann reaction negative in 5.

Relatives have goitre: 18 cases, 3 males. One patient was gravely anæmic, in 3 nasal disease was ascribed to exanthem. Wassermann reaction negative in 6. Only 1 patient had severe atrophy, 3 had crusting, all with fetor, and 3 others also had fetor; more than half the cases had excess of mucoid secretion, and none of these had fetor. Only 1 patient (A5: pus) had cacosmia.

Relationships in cases of goitre, hypothyroidism and rhinitis.
Cases of rhinitis are underlined.

FIG. 2.

This group shows a less direct connection with goitre, but is inserted owing to the frequent familial tendency in goitre, the assumption being that a defect which is expressed in one member of the family by the appearance of a goitre in another shows as nasal atrophy. In addition to those actually shown in this group many patients in the goitre group were relations, as mother and daughter, sisters, etc. The actual relationships noted are shown in Fig. 2. In no less than three instances we find grandmother with myxœdema (or hypothyroidism), mother with goitre, daughter with rhinitis. This group also affords strong evidence that the "goitre" type of rhinitis is a definite clinical entity, for in each of the three following cases the diagnosis "endocrine rhinitis" was made before the appearance of the goitre in a relative afforded confirmation :—

**Case 1.**—Female, aged 6 years. Seen early 1931 ; diagnosed "endocrine rhinitis" : A2, excess mucoid secretion, olfaction normal, complaining of "catarrh" ; no fetor, no cacosmia. Inquiry as to goitre in mother negative. Put on thyroid for eighteen months, then ceased treatment as "cured." Brought up for examination in January, 1934, by her father ; the child was quite well, but the father was seen to have a large goitre and typical rhinitis.

**Case 2.**—Female, aged 6 years. Seen March 1928 ; complaining of catarrh for two years : A3, crusting, fetor, Wassermann negative, antra normal ; no goitre in family. Ordered thyroid in January, 1929, for thirteen months, then "cured." Normal in March, 1933. Called up for examination January, 1934, and found normal ; brought with her her small sister *aged* 3 *with a large goitre*, but no nasal symptoms.

**Case 3.**—Female, aged 9 years. Came late in 1926 complaining of smell from the nose during two years : A3, crusting, anosmia, fetor, no cacosmia ; no goitre in family history. Put on iodine for four months ; improved, ceased to attend. Returned in January, 1933, with atrophy, but no fetor ; put on thyroid. January, 1934, no atrophy, no fetor, no symptoms ; came with her sister, who had been under treatment for goitre for four years.

Grouping together the last four sub-groups in which there is a more or less direct association of atrophy with goitre or other signs of endocrine mal-function, we have here a composite group of 131 cases, of whom only 13 are males. This is what one might expect in a condition associated with goitre. So, also, we might predict the large proportion of patients under thirty, which is still more conspicuous if the menopause group is neglected. Atrophy is rarely severe: the classification being mild in 23 cases, definite in 35, well developed in 63, and severe in only 8. One would expect the lesser degrees of atrophy to be the more common; possibly some of the milder cases are overlooked, but probably patients often do not come for advice until a certain stage has been reached. But no degree of atrophy that is seen in syphilitic or other groups is absent here. Rather over one-third of cases (37) have excess of mucoid secretion, only 5 have pus, 6 show crusting, and the remainder, over half, show only an abnormally dry mucous membrane (half of which in each sub-group have small scabs on the surface in addition). Olfaction is diminished or lost in one-third, the "dry" group being far the most affected, especially among those with the more advanced atrophy. The distribution of atrophy, crusting and other features between the fetid and non-fetid groups is shown below, the corresponding figures for the syphilitic and exanthem classes being shown for comparison. In the group we are now discussing the fetor is generally a faint "sourish-albuminous" smell with a tendency in some patients to be much more pronounced just before the menstrual period; it does not affect the "dry" more than the "moist." Where crusts or pus are found the severe fetor "typical" of atrophic rhinitis is usual. Cacosmia is commoner among the fetid than among the non-fetid (even though the former have nearly all lost the sense of smell), but is not specially prevalent with the extreme degrees of atrophy.

| | Syphilis. | | Exanthemata. | | Endocrine. | |
|---|---|---|---|---|---|---|
| | No fetor. | Fetor. | No fetor. | Fetor. | No fetor. | Fetor. |
| Cases .. .. .. | 42 | 83 | 5 | 19 | 101 | 32 |
| Extreme atrophy .. | 17 | 63 | 0 | 11 | 6 | 1 |
| Perforation .. .. | 5 | 37 | 0 | 2 | 0 | 2 |
| Crusting .. .. | 5 | 54 | 1 | 12 | 2 | 6 |
| Cacosmia .. .. | 11 | 47 | 1 | 8 | 8 | 10 |
| Anosmia .. .. | 23 | 67 | 0 | 0 | 9 | 28 |
| Excess mucus .. .. | 4 | 12 | 3 | 2 | 20 | 6 |

One may summarize the special features of this group of cases by saying that the patients are nearly all females, mostly below 30, although cases tend also to occur following the menopause. They complain usually of "catarrh" or dry throat, rather less frequently of deafness, fetor or simply of "goitre" without reference to the nose; occasionally of headache, nasal obstruction, etc. The usual findings are (1) a well-developed, but seldom severe, atrophy in which the lower part of the nose is chiefly affected, so that one can look right back into the naso-pharynx, while the middle turbinals stand out conspicuously near the septum in the upper part of the nose; (2) a nasal mucous membrane pale, thin and generally abnormally dry, often with small scabs on the surface, or flooded with thin, milky-watery mucoid secretion, only rarely showing crusts or pus; (3) about one-third of the patients exhibit fetor, which has usually a definitely "mucous" character, and is seldom severe except just before the menstrual period, but may be the typical offensive fetor known as "ozœna" if pus or crusts are present; (4) many of the patients with fetor are unconscious of it, having lost the sense of smell ; (5) the posterior wall of the pharynx is thin, often dry, and may show a film of drying mucus on the surface or a blob of thick mucus is extruded behind the soft palate when the patient gags ; (6) there may be other evidence of diminished endocrine activity ; (7) there is no evidence of syphilis, and the nasal sinuses are generally healthy ; (8) there is a rapid improvement on administering thyroid substance or iodine.

Thirty years ago and later a great deal of experimental and clinical work was done on the subject of ozœna (which was by many regarded as a definite disease), with a view to showing that a specific bacillary infection (*e.g.* with the bacillus of Perez) or an attenuated diphtheritic infection was the causative agent. The tendency for women to be chiefly affected, and for the condition to attack more than one member of a family was recognized, and we have shown above some evidence of the influence of diphtheria. But it is now generally admitted that the crusting and ozœna are the consequences and not the cause of the atrophy, and all my clinical observations go to support this thesis. In particular, exactly similar crusting, atrophy, and ozœna can be produced by the action of radium, or by injudicious surgical measures, especially removal of the inferior turbinal—the most certain means of inducing permanent atrophic rhinitis. There is, indeed, hardly any one clinical feature that can be called characteristic of "endocrine" atrophic rhinitis which is not seen also in syphilitic and post-exanthematous cases.

I may cite here the cases of two young women whom I treated for many weeks with glucose applications in 1922–23 ; they suffered severely from crusting and ozœna, but although temporarily benefited by the treatment, abandoned it as too tedious. One I saw again in 1929, the other in 1933 ; both had the same extreme atrophy, but in each case following the birth of a child, which presumably effected a profound change in endocrine functions, the crusting and fetor entirely disappeared.

This being so, the diagnosis must rest largely on the exclusion of syphilis, on the age of the patient, the general clinical picture described above, and particularly on the response to treatment. If there is also clear evidence of endocrine mal-function in the patient or her immediate relatives, this is strongly confirmatory. Perforation of the septum should make the investigation of syphilis very stringent ; on the other hand, atrophy in a child under ten or twelve should not be considered syphilitic without quite definite evidence elsewhere.

The diagnosis and treatment of these cases is important, particularly in the young, many of whom are sent up for tonsillectomy on account of "catarrh" or deafness, and complain of "sore" throat when only excessive dryness is meant. For not only does tonsillectomy not improve the symptoms, but the tendency to dryness is often accentuated by the operation, which is blamed for it—with a certain show of reason. I believe that the deafness is often not due to infection via the Eustachian tube, but is a consequence of participation in the atrophic process by the mucous membrane of the middle ear ; at least, in a number of cases this symptom has been relieved by treatment with thyroid gland substance without local treatment of the ear.

A number of patients have been classified as "similar to goitre" on the clinical appearances in the nose. This group consists of 55 patients, 19 males. There is no doubt that some of these ought to have been grouped with the "exanthem" group, and the features shown by the group as a whole are a blend of those of the exanthem and goitre groups. For example, crusting and severe atrophy are more common than one would expect with the latter. Other points are indicated in the table; this group has been included mainly because the similarity of clinical appearances led to treatment on the same lines as for the goitre group, and the results are worth recording for comparison.

The group described above as "endocrine rhinitis" is one in which there is (*a*) goitre or evidence of endocrine insufficiency, and (*b*) a tendency to nasal atrophy. By way of contrast a small group of cases has been separated in which the patients suffered from toxic goitre, Graves's disease or toxic thyroid adenoma; to these may be added 2 cases of myxœdema who had been taking large doses of thyroid for many years and 1 patient who came up complaining of dyspnœa—she was taking three grains of thyroid extract daily and was cured on halving the dose. There are 26 patients, 6 males. Rather more than half are over 30. Although there was excess of secretion in only 9 cases, yet of the whole 26, 9 patients showed *hypertrophy* of the nasal mucosa, 6 had no atrophy, 2 mild, 4 definite, 4 well-marked and only 1 severe atrophy. No case showed fetor, cacosmia, crusting, pus or perforation. In other words, this group shows an exactly opposite tendency in the nose towards excess function, and affords complementary evidence to support the association claimed between simple goitre and atrophy.

Before leaving the subject of clinical features a word must be said about infection of the nasal sinuses. It is remarkable how seldom these are infected, especially in the more severe cases of atrophy, although one would expect that with the nose full of abnormal secretions, pus or crusts their infection was almost certain. In the combined goitre group 29 cases were examined, only 7 were infected (and 4 of these had fetor). In the clinically similar group 18 cases were examined, 3 found infected (2 with fetor) That is, among 47 cases examined, which we may assume were practically all those in which the least likelihood of infection appeared, only 10 were infected, and among these it is probable that in 7 the sinus infection was responsible for the fetor. In 11 cases in all it was noted that the sinuses were remarkably smaller than normal ; whether this indicates some congenital abnormality, which may be part of an early failure in nasal development (and associated in this way with a tendency to nasal atrophy), or an early infective or other influence retarding development of sinuses and perhaps nose also, one cannot say.

*Treatment.*—A discussion of the causes of atrophic rhinitis would be merely of academic interest unless it led to a useful method of treatment. The treatment of this condition has been generally unsatisfactory. Some surgical methods aim at mechanical narrowing of the nasal passages by forcing together the maxillæ or by implanting cartilage into the turbinals, etc. The latter method has the defect that often it is difficult to discover anything but vestiges of the turbinals ; it was applied in 6 cases of the "goitre" and "similar" groups with a fair measure of success, and to 7 of the syphilitic with very little. Many methods of treatment aim either at stimulating the mucosa by local applications or merely at cleansing the nose of crusts and secretions; they succeed only as long as they are being applied, and are both tedious and comparatively unsatisfactory; nevertheless, they are the only resource in a large number of the worst cases.

During the seven years nearly all the cases of the "goitre" and "similar" groups have been treated by administration of iodine or thyroid by mouth. The iodine preparation usually favoured has been the "Liquor Iodi Simplex," from two to ten drops in milk or coffee twice or thrice daily; for a child of 10 five drops twice daily has been an average dose, for an adult thrice daily, but the dosage needs to be regulated to the clinical effect, and often has to be reduced after a few weeks. 28 patients were treated thus in addition to a few who had thyroid in addition, 2 who received potassium iodide, and 7 on Lugol's solution.

The dosage of thyroid has been even more varied, but most children of 10 can take half a grain daily of "Thyroid B.P. 1932" at least, and adults somewhat more; the aim has been to give as large a dose as can be taken without producing signs of excitement; several patients could not take even minute doses, but had to be treated with iodine. In the hypothyroid group 7 patients received "multigland" preparations and two thyroid with ovarian substance; including these, 110 patients were treated with thyroid (apart from those whose treatment has been too recently started for results).

Whichever treatment was adopted, regular clinical inspection has been made every two or four weeks, in most cases for at least six months, in many for three years. Children have responded better than adults. No nasal douche has been allowed, except in the few cases where there were actual crusts, and then only as long as these made douching necessary. In many cases where the nose was very dry an oily spray was ordered during the early stages. As a deodorant, and also where there was much excess of mucoid secretion, a snuff composed of menthol one part, lactic ferment nine parts, lactose ninety parts has been much appreciated — 3 patients received the snuff without other treatment. The results of treatment are shown in the table.

Of the 188 patients in these groups 17 have started treatment too recently for observation; 6 who were ordered treatment did not receive it; 110 were treated with thyroid gland, a few having iodine in addition; and 43 were treated with iodine in some form or other; the remainder were treated in other ways or received no treatment. Of the 153 treated with iodine or thyroid 19 are untraced, 34 are not improved and 7 are definitely improved but not cured; 93 are cured—most of these have been seen within the last three months, and it is fair to mention that a considerable number remain under treatment although "cured," a few (not re-examined) have reported that they remain free from all symptoms. The table shows the proportion of cures in various groups. Naturally this is greatest in the group with mild or trivial atrophy, but the proportions are not very dissimilar in the more advanced cases, and (to my astonishment) 6 of the 18 cases with originally severe atrophy are now without atrophy or other symptoms. It is curious that the "goitre" group shows the worst response to treatment, probably because fewer patients in this group were treated with thyroid gland than in the others. About sixty per cent. of all the patients in each sub-group who received thyroid, etc., have been relieved of all nasal symptoms: this supplies the most convincing support to the thesis that the nasal condition in these patients is directly associated with a defect in thyroid secretion.

## Figures and Tables

**Fig. 1. f1:**
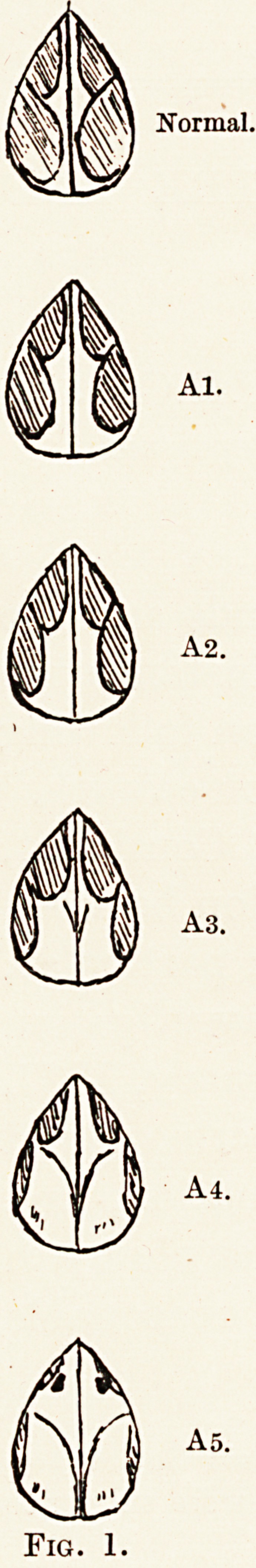


**Fig. 2. f2:**